# Androgen up-regulates vascular endothelial growth factor expression in prostate cancer cells via an Sp1 binding site

**DOI:** 10.1186/1476-4598-12-7

**Published:** 2013-02-01

**Authors:** Kurtis Eisermann, Carly J Broderick, Anton Bazarov, Mustafa M Moazam, Gail C Fraizer

**Affiliations:** 1 School of Biomedical Sciences, Kent State University, Kent, OH, USA; 2 Department of Biological Sciences, Kent State University, Kent, OH, USA; 3 Department of Pediatric Hematology/Oncology, Akron Children’s Hospital, Akron, OH, USA; 4 Department of Urology, University of Pittsburgh, Pittsburgh, PA, USA

## Abstract

**Background:**

Vascular Endothelial Growth Factor (VEGF) is regulated by a number of different factors, but the mechanism(s) behind androgen-mediated regulation of VEGF in prostate cancer are poorly understood.

**Results:**

Three novel androgen receptor (AR) binding sites were discovered in the *VEGF* promoter and *in vivo* binding of AR to these sites was demonstrated by chromatin immunoprecipitation. Mutation of these sites attenuated activation of the *VEGF* promoter by the androgen analog, R1881 in prostate cancer cells. The transcription factors AR and Sp1 were shown to form a nuclear complex and both bound the *VEGF* core promoter in chromatin of hormone treated CWR22Rv1 prostate cancer cells. The importance of the Sp1 binding site in hormone mediated activation of VEGF expression was demonstrated by site directed mutagenesis. Mutation of a critical Sp1 binding site (Sp1.4) in the *VEGF* core promoter region prevented activation by androgen. Similarly, suppression of Sp1 binding by Mithramycin A treatment significantly reduced *VEGF* expression.

**Conclusions:**

Our mechanistic study of androgen mediated induction of VEGF expression in prostate cancer cells revealed for the first time that this induction is mediated through the core promoter region and is dependent upon a critical Sp1 binding site. The importance of Sp1 binding suggests that therapy targeting the AR-Sp1 complex may dampen VEGF induced angiogenesis and, thereby, block prostate cancer progression, helping to maintain the indolent form of prostate cancer.

## Background

In the United States, prostate cancer is the most frequently diagnosed cancer in men with more than 200,000 new cases each year and the second most deadly, killing roughly 30,000 men annually [1]. Prostate cancer growth is dependent upon an adequate blood supply, which is controlled by Vascular Endothelial Growth Factor (VEGF), a regulator of tumor angiogenesis. Several factors are known to modulate VEGF expression including growth factors, cytokines, and hypoxia. Previous studies have also shown that androgen increases VEGF levels [2-5], but the mechanism(s) involved are unknown.

The *VEGF* promoter lacks a TATA box, is GC rich, and is regulated by multiple transcription factors, such as AP-2, HIF-1, Egr1, and WT1 [6-10]. Previously we have reported the identification of functional WT1 binding sites within the proximal *VEGF* promoter [7,11], and others have reported interaction of WT1 and HIF1-α in the regulation of VEGF [8]. Additionally, Sp1/Sp3 binding sites located in the core promoter are known to play a role in transcriptional regulation of *VEGF* in a variety of cell lines including NIH3T3 cells [12], ZR-75 breast cancer cells [13], Y79 retinoblastoma cells [14], NCI-H322 bronchioloalveolar cells [15], and PANC-1 pancreatic cells [16]. Members of the Sp family have a conserved C-terminal DNA binding domain, so they can potentially bind the same sequence of DNA and indeed Sp1, 3, and 4 bind preferentially bind at GC-boxes [17]. However, binding at different sites within a promoter region may also confer different functional responses for Sp1 and Sp3 [18]. A cluster of Sp1/3 sites in the proximal promoter mediates regulation of VEGF by TNF-α in human glioma cells [19]. Sp1/3 sites are also required for IL-1β induction of *VEGF* transcription in cardiac myocytes [20] and for TGF-β1 stimulation of *VEGF* transcription in cholangiocellular carcinoma cells [21]. In Panc-1 pancreatic cells, the regulation of VEGF by Sp1 has been extensively documented [16,22] and both constitutive Sp1 activity and a 109 bp core promoter region containing Sp1 sites are essential for VEGF expression [16]. Overall, the transcriptional regulation of *VEGF* is cell specific involving different stimuli and factors, but Sp1 plays a prominent role in many cell types.

Since estrogen mediated regulation of VEGF expression in ZR-75 breast cancer cells was shown to require Sp1 sites in the core *VEGF* promoter [13], we asked whether androgen might behave similarly in prostate cancer cells. Previous studies have demonstrated that *VEGF* mRNA levels are elevated by androgen treatment of both human fetal prostatic fibroblasts and LNCaP prostate cancer cells [2,4,5]. Also, VEGF protein levels are increased after treatment with hormone [3] and flutamide, an anti-androgen, has been shown to block this up-regulation [23]. However, the hormone responsive region of the *VEGF* promoter was never identified in these earlier studies, nor was the mechanism of androgen induction of *VEGF* promoter activity and *VEGF* mRNA expression determined.

This report characterizing the hormone responsive regions and binding sites within the *VEGF* promoter is a continuation of earlier studies analyzing conserved putative binding sites in promoters of genes expressed in prostate cancer [11] that identified potentially important non-classical AR binding sites adjacent to other zinc finger transcription factor binding sites in the promoter of *VEGF* and other genes [24]. Here we identified and characterized the hormone responsive regions of the *VEGF* promoter, including a required Sp1 binding site within the core promoter.

## Results

### Androgen induces VEGF expression and AR binding to chromatin of prostate cancer cells

**Figure 1:**
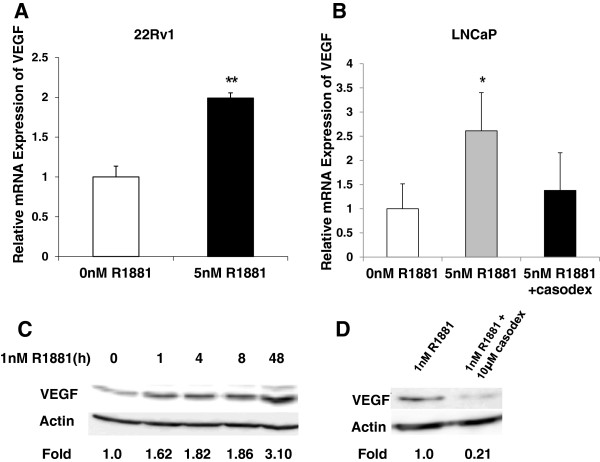
**Androgen regulates VEGF expression in LNCaP and 22Rv1 prostate cancer cells.** (**A**) 22Rv1 cells were serum starved overnight then treated with 5nM R1881 or DMSO as a vehicle control (0nM R1881). *VEGF* mRNA expression was measured by qRT-PCR and normalized by β-actin levels as described in text. (**B**) *VEGF* mRNA expression in LNCaP cells was measured by qRT-PCR and normalized by 18S levels, as described in text. Cells were serum starved as described in A. For inhibition of androgen activity, cells were pre-treated with 10μM casodex for 2 hrs and then induced with 5nM R1881 for 24 hrs. Values represent fold change relative to DMSO treatment. A Student’s t-test was performed and significance was determined * (p < 0.05), ** (p < 0.01). (**C**) VEGF protein expression in LNCaP cells treated with 1nM R1881 for 0–48 hours. Protein expression was measured by western blotting as described in text, and β-actin levels were used as loading controls. (**D**) Cytoplasmic VEGF protein expression was measured by western blot of LNCaP cells treated as per (B). Image J analysis was performed and VEGF levels were normalized to β-actin levels. Shown are relative fold-changes in VEGF protein levels, normalized to β-actin and relative to untreated cells.

To determine whether VEGF expression was activated by androgen in prostate cancer cells, CWR22Rv1 (22Rv1) cells were treated with the androgen analog R1881. Cells were serum starved overnight and then treated with 5nM R1881 for 48 hours. Figure 1A shows a two-fold increase in *VEGF* mRNA expression in response to androgen, as measured by quantitative real-time PCR (qRT-PCR). Similar effects were observed in LNCaP cells treated with 1nM R1881 (Additional file 1) and 5nM R1881 (Figure 1B). To confirm that androgen induction of VEGF required hormone-AR interaction, the effect of anti-androgen treatment was then examined using bicalutamide (casodex). LNCaP cells were pre-treated with 0 μM or 10μM casodex for 2 hrs and then treated with 5nM R1881 for 24 hours. Casodex treatment significantly reduced the hormone activation of *VEGF* mRNA indicating that classical signaling requiring AR-androgen interaction was occurring (Figure 1B). Inhibition of hormone induced *VEGF* expression by casodex was confirmed in 22Rv1 cells (data not shown). Given that hormone enhanced VEGF mRNA levels, VEGF protein expression was also examined in LNCaP cells treated with androgen. As shown in Figure 1C, VEGF protein expression increased after 1 hour of treatment with 1nM R1881 and maximal expression was seen after 48 hours, which was similar to mRNA expression. Blockade of classical androgen signaling by casodex treatment also decreased hormone mediated up-regulation of cytoplasmic VEGF protein levels by more than 70%. (Figure 1D). To determine whether casodex also blocked a hormone mediated increase in nuclear AR protein levels, nuclear extracts were isolated and western blot analysis was performed. AR protein levels in nuclear lysates prepared from LNCaP cells treated with 0nM or 1nM R1881 and with 0μM or 10μM casodex were examined, and casodex was shown to significantly reduce AR protein levels (data not shown).

**Figure 2:**
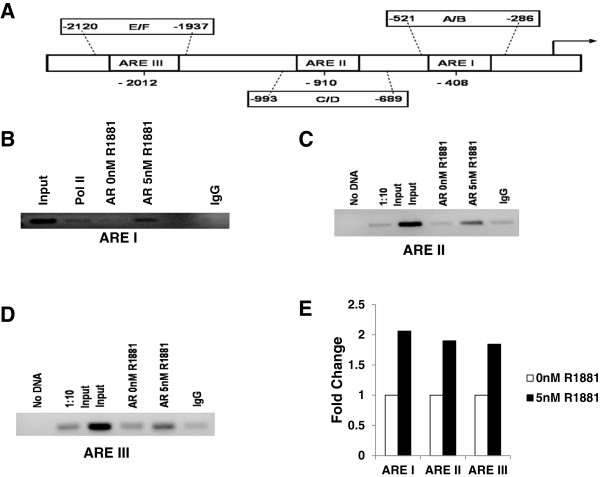
**Hormone treatment enhances AR protein binding to the *VEGF *promoter in chromatin of LNCaP cells.** (**A**) Schematic diagram of the *VEGF* promoter showing the location of predicted ARE binding sites and primers used to amplify the specific regions of the promoter. (**B**) ChIP assays were performed with primers specific for the ARE I region of the *VEGF* promoter using chromatin prepared from LNCaP cells treated for 24 hours with either 0nM R1881 or 5nM R1881, following overnight serum starvation. Standard endpoint PCR was performed as described in text. Lane 1 shows amplification of input chromatin that was not immunoprecipitated with antibody, lane 2 chromatin immunoprecipitated with anti-pol II antibody (Upstate), lanes 3 and 4 chromatin from cells treated with 0nM or 5nM R1881 and immunoprecipitated with anti-AR (Santa Cruz) antibody and lane 6 is the negative control precipitated with IgG (Upstate). Chromatin amplified in lane 3 was obtained from cells treated with vehicle (DMSO) only. (**C**) PCR was performed using chromatin as described in (B) and primers specific for the ARE II region (shown in A). Lane 1 is the no DNA control, lane 2 is input diluted 1:10, lane 3 is undiluted input, lanes 4 and 5 chromatin from cells treated with 0nM or 5nM R1881 and immunoprecipitated with anti-AR antibody, and lane 6 is the IgG negative control precipitation. Chromatin amplified in lane 4 was obtained from cells treated with vehicle (DMSO) only**.** (**D**) PCR was performed using chromatin as described in (B) and primers specific for the ARE III region. Lanes are the same as in (C). (**E**) Quantification of immunoprecipitation was performed by SYBR Green qRT-PCR using primers described in (A). Chromatin was immunoprecipitated with anti-AR antibody from LNCaP cells treated with 0nM or 5nM R1881 as described above. Average Ct values of immunoprecipitated chromatin were normalized to input and normalized values from 5 nM R1881 treated cells are shown relative to untreated cells (0nM R1881).

Having confirmed classical hormone mediated VEGF up-regulation, potential ARE binding sites within the *VEGF* promoter were then identified using MatInspector software, as previously described [24]. Transcription factor binding site prediction analysis of the *VEGF* promoter sequence revealed numerous transcription factor binding sites including Sp1, WT1, and Egr1 sites as well as three potential ARE binding sites within 2kb of the transcription start site in the *VEGF* promoter. Since these ARE sites (Figure 2A) were non-classical monomeric sites, it was important that they be tested for functional binding using chromatin immunoprecipitation (ChIP). LNCaP cells were serum starved overnight and then treated with 0nM or 5nM R1881 for 24 hours. As shown in Figures 2B-D**,** chromatin of hormone treated LNCaP cells was immunoprecipitated with anti-AR antibody and amplified by three primer sets flanking the regions containing the three putative ARE binding sites (Figure 2A). Hormone treatment enhanced AR binding, as indicated by both standard endpoint PCR (Figures 2B-D) and SYBR Green quantitative qRT-PCR (Figure 2E). Results were quantified as a percentage of input chromatin and showed approximately 2-fold increase of chromatin immunoprecipitated by AR antibody in cells treated with 5nM R1881 compared to that of untreated cells (Figure 2E). These results suggested that all three binding sites were functional and might be important in the hormone regulation of *VEGF*.

### Three non-classical ARE sites contribute to the hormone response of the *VEGF* promoter

**Figure 3:**
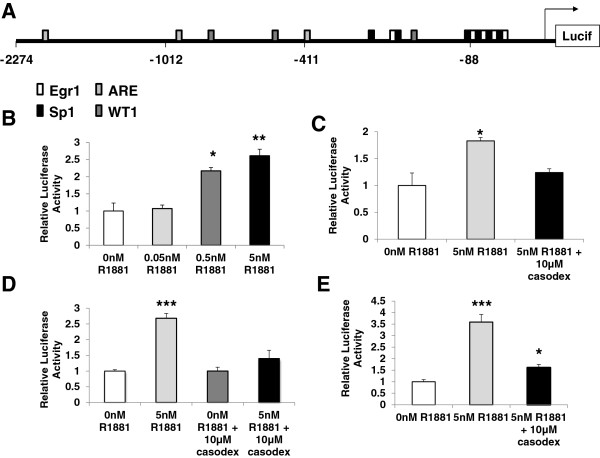
**Hormone activates the *VEGF *promoter in two different cell lines, LNCaP and 22Rv1.** (**A**) Schematic diagram of the *VEGF* promoter showing locations of predicted ARE binding sites (grey boxes) in relation to 5’ termini of luciferase reporter constructs V411 and V2274 [16]. (**B**) 22Rv1 cells were transfected with the 411 bp *VEGF* promoter construct (V411) in the presence or absence of different concentrations of R1881 (0, 0.05nM, 0.5nM, and 5nM) for 48hrs. Luciferase assays of cell lysates were performed and fold activation was determined as described in text. (**C**) 22Rv1 cells were transfected with V411 and treated with 0nM R1881, or 5nM R1881 with 0μM casodex, or 5nM R1881 with 10μM casodex for 48 hrs. (**D**) LNCaP cells were transfected and treated as per (C). (**E**) 22Rv1 cells were transfected with the 2274 bp *VEGF* promoter construct (V2274) and treated as per (C). Luciferase activity is shown relative to average normalized luciferase activity in the absence of hormone. Experiments were repeated three times in triplicate. Significance was determined by Student’s t-test (*p<0.05, ** p<0.01, ***p<0.001).

To determine whether AR binding regions identified by ChIP were transcriptionally activated by hormone, a series of *VEGF* promoter deletion constructs were obtained [16] ranging in length from 88 bp (V88) to 2274 bp (V2274). Figure 3A shows the location of predicted Sp1, WT1, Egr1, and AR transcription factor binding sites within the 2kb promoter region. These constructs were tested in luciferase assays to determine where within the *VEGF* promoter the hormone responsive element(s) were located. LNCaP and 22Rv1 cells were transfected with a 411 bp (V411) construct containing only the ARE I site and treated with either increasing doses of R1881 (0.05 to 5nM) (Figure 3B**)** or 5nM R1881 with 10μM casodex (Figure 3C and D). After 48 hours, cells were lysed and luciferase assays were performed. 22Rv1 cells were shown to be highly sensitive to androgen as even 0.5nM R1881 increased *VEGF* promoter activity more than 2 fold (Figure 3B). Similarly, in 22Rv1 cells an almost 2 fold increase in *VEGF* promoter activity was seen in cells treated with 5nM R1881 and casodex blocked this activation (Figure 3C). Additionally, LNCaP cells treated with 5nM R1881 showed a greater than a 2.5 fold increase in *VEGF* promoter activity when compared to cells treated with the DMSO vehicle control (Figure 3D). Confirming the requirement for AR-hormone interaction, casodex treatment inhibited this androgen response in LNCaP cells (Figure 3D). Since ARE II and III lie outside of the V411 region, a larger promoter construct (V2274) was examined in 22Rv1 cells to determine whether hormone activation of *VEGF* was greater in the 2kb reporter construct containing all three ARE binding sites. As shown in Figure 3E, hormone activation of V2274 was increased (3.5 fold) in this larger construct, greater than the response shown in the smaller 411 bp reporter construct containing only ARE I. This suggested that all three ARE sites may contribute to androgen activation of the *VEGF* promoter, although not synergistically.

**Figure 4:**
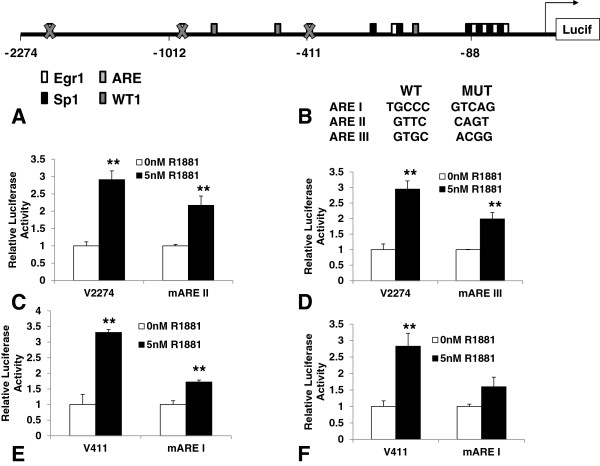
**Mutation of ARE sites attenuate hormone activation of the *VEGF *promoter.** (**A**) Schematic diagram of a 2kb region of the *VEGF* promoter showing the ARE I-III binding sites. Thick X’s indicate sites that were mutated. (**B**) Shown are the wild type (WT) and mutant (MUT) sequences of the ARE I, ARE II, and ARE III binding sites. Site-directed mutagenesis was performed as described in text, using primers containing these mutated bases. (**C**) Wild type V2274 and mutant (mARE II) luciferase reporters were transfected into 22Rv1 cells treated with 0nM or 5nM R1881 for 48 hrs. Luciferase assays were performed as previously described. (**D**) Mutant (mARE III) and wild type V2274 were transfected into 22Rv1 cells as in (C). (**E**) Wild type and mutant V411 constructs were transfected into 22Rv1 cells as in C. (**F**) The mutant V411 construct was also transfected into LNCaP cells and treated as in C. Luciferase activity is shown relative to average normalized luciferase activity in the absence of hormone. Experiments were repeated three times in triplicate. Significance was determined by Student’s t-test (**p<0.01).

To determine which ARE sites might be required for androgen mediated up-regulation of the *VEGF* promoter, all three ARE binding sites were mutated and mutations were confirmed by sequence analysis. Mutations were initially made in the larger V2274 reporter construct which contains all three ARE binding sites (Figure 4A). Site-directed mutagenesis was performed using PCR primers designed to contain base substitutions in either the ARE II or ARE III sites in the V2274 construct (Figure 4B). The effect of eliminating ARE binding at the ARE II or III sites was tested by luciferase reporter assays performed in 22Rv1 cells. Transfections of 22Rv1 cells were followed by hormone treatment and the wild type V2274 promoter construct was up-regulated approximately 3 fold by 5nM R1881 and this response was attenuated in the mutant constructs to approximately 2 fold activation (Figures 4C and D). Double mutation of both ARE II and III sites in V2274 showed similar retention of residual hormone activation when compared to wild type (data not shown). To determine the contribution of ARE I, the V411 reporter construct containing only the ARE I site was mutated as described (Figure 4B) and the effect was tested by luciferase reporter assays performed in both 22Rv1 and LNCaP cells. Figure 4E shows that in 22Rv1 cells, the wild type V411 promoter was up-regulated more than 3 fold by 5nM R1881 and this response was attenuated in the mutant ARE I V411 construct to less than 2 fold activation. A similar effect was also seen in LNCaP cells (Figure 4F), although in this case the residual hormone response of the mutant ARE I -V411 construct was not significant. Overall the hormone response of the mutant ARE I promoter was reduced approximately 2-fold in both LNCaP and 22Rv1 cells.

Since single or double mutation of the three ARE sites did not completely eliminate hormone response, we reasoned that one possibility was that all three sites were redundant. However, mutation of two of three ARE sites did not reduce hormone activation to any greater extent than one site alone (data not shown). Another possibility was that other TFs were involved in the hormone response. Thus, we examined the involvement of Sp1, another ZFTF known to regulate VEGF transcription in other systems.

### Sp1 binding site in the *VEGF* core promoter is required for hormone responsiveness

**Figure 5:**
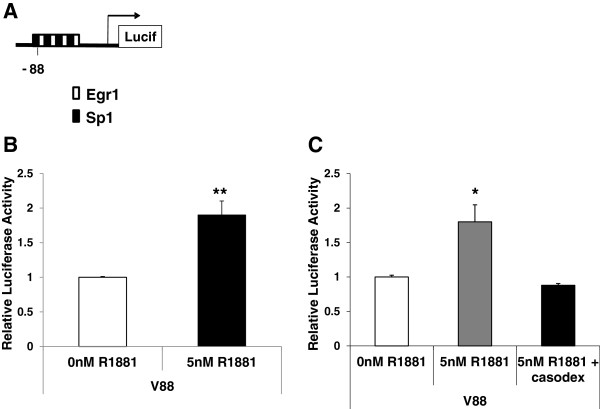
**Hormone activates the *VEGF *minimal core promoter region lacking ARE sites.** (**A**) Schematic diagram of an 88bp region of the *VEGF* core promoter showing four predicted Sp1 (black box) and Egr1 (white box) binding sites. (**B**) 22Rv1 cells were transfected with V88 followed by treatment with 0nM or 5nM R1881 as described above. Luciferase assays were performed as previously described. (**C**) 22Rv1 cells were transfected as in (B) except pre-treated with 0μM or 10μM casodex for 2 hours. Luciferase activity is shown relative to average normalized luciferase activity in the absence of hormone. Experiments were repeated three times in triplicate. Significance was determined by Student’s t-test (*p<0.05, ** p<0.01).

Given that potential Sp1 binding sites were also identified in the *VEGF* promoter region and Sp1 was observed to bind the *VEGF* promoter in chromatin of LNCaP cells [11], we asked whether Sp1 binding might contribute to androgen activation of the *VEGF* promoter. Although the V88 core promoter region is a very G-rich region, containing multiple potential Sp1 binding sites (Figure 5A), it was not expected to respond to hormone induction, as it does not contain any ARE binding sites. Initially, to test the hormone response of the core promoter, 22Rv1 cells were co-transfected with the V88 reporter construct followed by treatment with R1881. Luciferase assays were performed and surprisingly androgen activated this *VEGF* core promoter region almost 2-fold (Figure 5B). To confirm this hormone activation was mediated by AR/androgen interaction, V88 transfected 22Rv1 cells were treated with casodex. Figure 5C shows that casodex treatment completely blocked the androgen activation of V88, implicating classical androgen signaling at the GC-rich core promoter.

**Figure 6:**
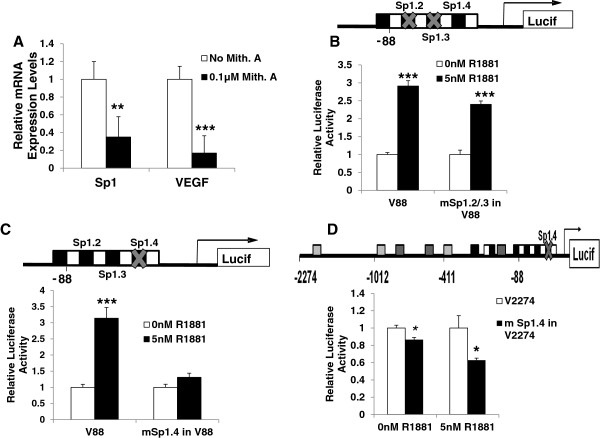
**Inhibition of Sp1 binding attenuates hormone activation of the *VEGF *core promoter.** (**A**) 22Rv1 cells were treated with DMSO or 0.1μM Mithramycin A for 24 hours. *Sp1* and *VEGF* expression were measured by SYBR Green qRT-PCR using primers specific for *Sp1* and *VEGF* and normalized to *GAPDH*. Values represent fold change relative to cells treated with DMSO vehicle control. (**B**) Schematic diagram of the 88bp region of the *VEGF* core promoter (as shown in Figure 5). Gray X’s indicate locations of two mutations created at the Sp1.2 and Sp1.3 binding sites, as described in the text. 22Rv1 cells were transfected with wildtype V88 or dual mutant (mSp1.2/.3) constructs followed by treatment with 0nM or 5nM R1881 as described above. (**C**) The Sp1.4 binding site in the V88 reporter construct was mutated (shown as above) and 22Rv1 cells were transfected as described for (B). (**D**) Sp1.4 binding site was mutated in the V2274 reporter construct and transfected into 22Rv1 cells treated with 0 or 5nM R1881 for 48 hours. Luciferase assays were performed as described previously. Luciferase activity is shown relative to average normalized luciferase activity in the absence of hormone. Experiments were repeated at least twice in triplicate. Significance was determined by Student’s t-test (* p<0.05, ** p<0.01, and *** p<0.001).

Since the core promoter contains multiple Sp1 binding sites and Mithramycin A is an inhibitor of transcription factor binding to GC rich promoters, we treated 22Rv1 cells with 0.1μM of Mithramycin A for 24 hours to block Sp mediated activation of the *VEGF* promoter. RNA was isolated and qRT-PCR was performed to determine the effect on *VEGF* mRNA levels. Figure 6A shows that, as expected, Mithramycin A treatment suppressed *VEGF* expression as well as *Sp1,* itself. *VEGF* mRNA levels were repressed more than 5 fold, and *Sp1* more than 2.5 fold, in the presence of Mithramycin A. To confirm the necessity of Sp1 binding, site-directed mutagenesis was used to mutate three potential Sp1 binding sites within the core promoter. Primers were designed incorporating mutations for both the Sp1.2 and Sp1.3 binding sites as shown (Figure 6B). Hormone treatment significantly activated the core promoter containing mutant Sp1.2/.3 binding sites (2.5 fold) as demonstrated by luciferase assays of R1881 treated 22Rv1 cells (Figure 6B). This suggested that these two potential Sp1 binding sites were not essential for hormone response, so another Sp1 binding site (Sp1.4) closer to the transcriptional start site was examined. Mutation of this site completely suppressed activation by R1881 (Figure 6C). That is, wild-type core promoter was activated by hormone treatment 3 fold, whereas the mSp1.4 mutant core promoter was not significantly activated (1.3 fold increase). This reduction of hormone response suggested that the Sp1.4 site was required for a full-strength response of the core promoter to R1881.

To address the possibility that mutation of the Sp1.4 site resulted in a loss of both basal *VEGF* expression and the induced hormone mediated response we asked whether the mutation altered basal activity. If Sp1 binding at the Sp1.4 site was required for basal transcription, then we would predict both hormone and basal response would be reduced by the mutation. Therefore we compared the basal expression levels of the mutant mSp1.4 promoter construct to the empty vector and found that there was still significant basal transcription occurring (Additional file 2). This data suggests that while the Sp1.4 binding site was essential for hormone response, it was not required for basal activity. Since mutation of Sp1.4 in the V88 construct eliminated androgen induction of the core promoter, it was important that this binding site also be mutated in the V2274 promoter construct to confirm that the Sp1.4 binding site regulated hormone activation of the full length promoter region, which contains all three ARE binding sites. This was indeed the case, as mutating Sp1.4 within the V2274 construct decreased induction by R1881 (Figure 6D). This was consistent with Figure 6A, which demonstrated transcriptional suppression of the endogenous *VEGF* promoter by Mithramycin A, an inhibitor of Sp1 binding. Overall the data demonstrated the critical role of the Sp1.4 binding site for hormone mediated activation of *VEGF* expression.

### AR and Sp1 interact in the nucleus and both bind the *VEGF* core promoter in chromatin

**Figure 7:**
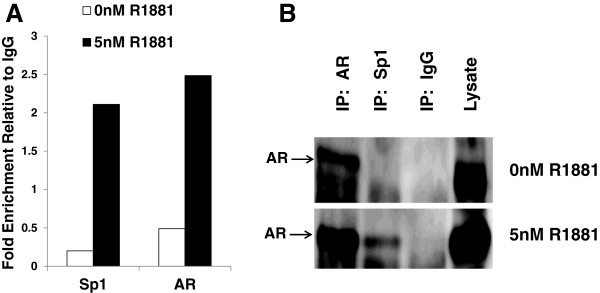
**AR and Sp1 form a complex and bind to the VEGF core promoter.** (**A**) Quantitation of AR and Sp1 immunoprecipitated chromatin was performed by qRTPCR amplification of the *VEGF* core promoter region following ChIP assay of 22Rv1 cells treated with 0nM or 5nM R1881 for 48 hours prior to harvest, as described in Figure 2. Chromatin was immunoprecipitated with AR (Santa Cruz) or Sp1 (Upstate) antibodies, or IgG (Upstate), then quantified by SYBR-Green qRT-PCR using primers specific for the *VEGF* core promoter region (as described in text). Values shown are fold enrichment of Ab specific immunoprecipitations compared to IgG controls. (**B**) The interaction of AR and Sp1 in hormone treated 22Rv1 cells was demonstrated by co-immunoprecipitation of cells treated with 0nM or 5nM R1881 for 48 hours prior to harvest. Nuclear extracts were incubated with either AR Ab (Santa Cruz), Sp1 Ab (Upstate), or serum IgG and the complexes were collected by sepharose magnetic beads (Active Motif, CA). Proteins eluted from the beads were denatured and electrophoresed, then blots were probed with AR Ab. Top and bottom panels show proteins immunoprecipitated from cells treated with 0nM or 5nM R1881, respectively.

In addition to these reporter assays showing that the Sp1.4 binding site was required for androgen mediated up-regulation of the *VEGF* promoter, prior electrophoretic mobility shift assays (EMSA) of the *VEGF* core promoter had also shown that an oligonucleotide sequence containing the Sp1.4 site bound purified Sp1 proteins *in vitro*[7]. Here, we determined whether Sp1 would bind the *VEGF* promoter *in vivo.* ChIP analysis was performed to determine if both Sp1 and AR bound to the GC- rich core *VEGF* promoter in chromatin of hormone treated 22Rv1 cells (Figure 7A). Sp1 and AR immunoprecipitated chromatin was quantified by qRT-PCR and results clearly show that both Sp1 and AR bound to this GC-rich region**.** In cells treated with 5nM R1881, 10-fold more chromatin was immunoprecipitated by Sp1 antibodies than in untreated cells. Similarly, hormone treatment increased AR binding to this region by more than 5 fold, despite the absence of ARE sites. These results indicate that both AR and Sp1 were bound to the core *VEGF* promoter region and suggested the possibility that they might form a complex tethering AR to Sp1 binding sites within the core promoter.

Although AR and Sp1 have previously been demonstrated to interact in LNCaP cells [25], it was unclear whether these two zinc finger transcription factors would form a complex in 22Rv1 cells. Nuclear lysates from 22Rv1 cells were immunoprecipitated using AR and Sp1 antibodies as well as a negative control IgG. Using Western blot analysis, the immunoprecipitated proteins were then probed with AR antibody. In the absence of hormone, little AR protein was precipitated by AR antibody and none by Sp1 antibody (Figure 7B**,** upper panel). In contrast, in the presence of 5nM R1881, AR protein was immunoprecipitated by both Sp1 and AR antibodies (Figure 7B**,** lower panel). This co-immunoprecipitation was hormone specific, occurring only in cells that were treated with 5nM R1881, indicating that Sp1/AR complex formation was an androgen dependent interaction. This hormone dependent complex formation is consistent with hormone mediated binding and activation of the *VEGF* core promoter.

## Discussion

We and others have previously demonstrated that androgens up-regulate VEGF expression [3,4,7,23], however, mechanisms involved were not elucidated. Therefore, a molecular understanding of how androgens regulate VEGF in prostate cancer cells was sought. In this study, we firmly established *VEGF* as a hormone responsive gene and demonstrated that the Sp1.4 binding site located 50 bp downstream of the transcription start site of *VEGF* was necessary for androgen activation of the *VEGF* core promoter, a region lacking any potential ARE binding sites, yet responsive to hormone treatment. Consistent with this finding was the observation that Mithramycin A, which inhibits Sp1 binding to GC rich promoter regions, significantly decreased *VEGF* mRNA levels. Sp1 mediated hormone activation of the *VEGF* core promoter likely involves both DNA binding and AR protein interaction, as demonstrated by ChIP and co-immunoprecipitation assays. These results support a tethering model for hormone activation of the *VEGF* core promoter, i.e., that ligand-bound AR is recruited and then held in place by chromatin-bound Sp1 at the core promoter.

In delineating the hormone responsive regions of the *VEGF* promoter, we initially focused on three potential ARE sites and demonstrated DNA binding by ChIP and transcriptional activation by R1881. However, mutations of the three functional ARE half-sites merely attenuated, but did not eliminate activation of *VEGF* by androgen. Androgens are known to regulate a multitude of genes, with the most well studied androgen regulated gene being *PSA*. There are three known dimeric ARE binding sites in the regulatory region of *PSA*[26] and, similar to the *VEGF* promoter, all three sites are involved in androgen regulation. Although mutation of the two AREs in the proximal promoter of *PSA* significantly decreased activation by R1881, mutation of the ARE in the distal enhancer located 4kb upstream of the transcription start site completely blocked androgen activation of *PSA*. In contrast to *PSA*, the ARE sites in the *VEGF* promoter are monomeric sites, not canonical dimeric ARE sequence, and are located within 2 kb of the start site. One possibility is that binding at these non-classical sites in the *VEGF* promoter may not be as strong as that in the *PSA* promoter, but interaction with other TFs might enhance or stabilize binding of AR, increasing *VEGF* expression.

Since the *VEGF* promoter region is highly GC-rich we investigated the role of other zinc finger transcription factors known to bind GC-rich promoter regions, such as Sp1. Sp1/Sp3 binding sites in the core promoter region are known to control *VEGF* transcriptional regulation in a number of different cell lines. Sp1 mediates regulation of VEGF in the presence of specific stimuli, such as stress [27], estrogen [13], retinoic acid [14], TGF-β1 [21], and PDGF [12] depending on the cell type. Androgens have been known to act in concert with other zinc finger transcription factors such as GATA [28] and Sp1 [25,29] to regulate androgen responsive genes such as *PSA*, *p21*, and *NRIP*. Previously, Sp1 sites have been shown to be involved in androgen induction of both the *p21* gene and the *NRIP* (*nuclear receptor interaction protein*) gene and co-IP demonstrated that AR interacts with Sp1 to regulate their expression [25,29]. Similarly we have demonstrated that Sp1 plays a role in the androgen responsiveness of *VEGF* by forming a complex with AR and binding to the *VEGF* promoter. While there are four Sp1 binding sites in the core promoter region, mutation of a single binding site, Sp1.4, eliminated androgen induction of this region of *VEGF*. The *VEGF* promoter is similar to the *NRIP* promoter, in that both are TATA-less GC rich promoters that are induced by androgen in prostate cancer cells [29]. The *NRIP* promoter also contains three Sp1 sites and two hormone responsive elements (ARE and GRE). Similar to our findings in the *VEGF* promoter, mutation of these ARE/GRE sites did not eliminate hormone response; and Sp1 and AR were shown to cooperatively interact by several methods, including sequential chromatin immunoprecipitation and co-IP. In both these promoters, the association of AR with Sp1 appeared to cooperatively regulate promoter activity.

The mechanism of androgen mediated regulation of *VEGF* identified by this study is analogous to estrogen mediated regulation of *VEGF* in breast cancer cells [13]. We show here that androgen up-regulates the *VEGF* core promoter, a region lacking ARE binding sites, but containing four binding sites in which Sp1 or Sp3 can bind. In ZR-75 breast cancer cells, estrogen regulation of *VEGF* expression is thought to act through ER- α/Sp1 and ER- α/Sp3 interactions with GC-rich motifs [13]. These authors showed that treatment with estradiol increased *VEGF* mRNA levels greater than fourfold. Additionally, the GC-rich region of the *VEGF* core promoter (−66 to −47) was required for E2 activation of *VEGF*, despite a lack of classical ER binding sites. Both Sp1 and Sp3 were demonstrated to bind the *VEGF* promoter *in vitro* by EMSA and *in vivo* by ChIP, further supporting their functional relevance in E2-mediated regulation of *VEGF*. While these non-classical mechanisms of hormone mediated *VEGF* regulation operate under normoxic conditions, under hypoxic conditions HIF-1α is known to regulate *VEGF* expression. Androgen regulation of *VEGF* by HIF-1α is thought to occur indirectly through an autocrine loop involving EGF/phosphatidylinositol 3^′^-kinase/protein kinase B, which activates HIF-1α and HIF-1α regulated expression of *VEGF* under hypoxic conditions [23].

## Conclusions

Androgen mediated regulation of *VEGF* expression required a specific Sp1/3 binding site in the GC-rich *VEGF* core promoter. Although ARE sites within the *VEGF* promoter bound AR and their mutation dampened *VEGF* expression, mutation of a key Sp1 binding site in the core promoter of *VEGF* blocked promoter activation by hormone. Our findings with androgen reflect those of others examining regulation of *VEGF* by other hormones [13-15]; overall these studies demonstrate the complexity of hormone activation of *VEGF* and the importance of protein-protein interactions. Regulation of *VEGF* by zinc finger transcription factors, such as Sp1, and the importance of their interactions with AR, suggests that they may play a positive role in promoting angiogenesis and prostate cancer progression. Thus, elevated expression of these zinc finger transcription factors may indicate a worse prognosis. Therapy disrupting AR-Sp1 complexes and thereby suppressing VEGF would be expected to limit angiogenesis and maintain the indolent form of prostate cancer.

## Methods

### Cell culture and hormone treatment

LNCaP (ATCC CRL-1740) and CWR22Rv1 (ATCC CRL-2505) prostate cancer cells were cultured in RPMI media. All cells were grown in media supplemented with 10% FCS and 100ug/ml penicillin/streptomycin in a 37°C incubator with 5% CO_2_. For hormone treatment, cells were grown to 60-80% confluency and then serum starved overnight in either serum-free media or media supplemented with 5% charcoal-dextran stripped FBS RPMI. The synthetic androgen methyltrienolone (R1881) (Perkin Elmer, Boston, MA) was then added to the charcoal-dextran stripped FBS RPMI media and cells were treated with 5nM R1881 for 24 hours unless otherwise noted in figure legends. For inhibition of AR, 10μM bicalutamide/casodex (LKT Labs, St. Paul, MN) was added 2 hours prior to treatment with R1881.

### Chromatin immunoprecipitation

Two million cells were treated with formaldehyde to crosslink proteins to DNA and lysed as per manufacturer’s recommendations using Millipore EZ ChIP Assay (Upstate Biotechnology Inc., Billerica, MA). Chromatin was sheared by sonication (Biosonik III, Bronwill Scientific, Rochester, NY) to fragments of 200–1,000 bp in length. The supernatant was pre-cleared by incubation with Protein G Agarose and incubated overnight at 4°C with either anti- AR (Santa Cruz), Sp1 (Santa Cruz) and control polymerase II antibodies or non-immune IgG (Upstate Biotechnology Inc.). The complexes were recovered from Protein G magnetic beads, crosslinks were reversed and DNA was purified. Four percent of both immunoprecipitated and input chromatin were amplified by PCR using *Taq* polymerase (Applied Biosystems by Roche Molecular System, Inc) and the appropriate primers (ARE I (FOR): 5^′^-TTCGAGAGTGAGGACGTGTG-3^′^, ARE I (REV): 5^′^-AGGGAGCA GGAAA GTGAGGT-3^′^, ARE II (FOR): 5^′^-TCACTGACTAACCCCGGAAC-3^′^, ARE II (REV): 5^′^-TTTGG GACTGGAGTTGCTTC-3^′^, ARE III (FOR): 5^′^-GGCTCTTTTAGGGGCTGAAG-3^′^, ARE III (REV): 5^′^-AGGCTGATGAACGGGATATG-3^′^, VEGF V88 (FOR) 5^′^-CCGCGGGCGCGTGTC TCTGG-3^′^, VEGF V88 (REV) 5^′^-TGCCCCAAGCCTCCGCGATCCTC-3^′^). Following an initial 10 min denaturation at 95°C, DNA was amplified by 32–35 cycles of: 1) 20 sec denaturation at 95°C, 2) 30 sec annealing at either 52°C (for ARE III primers), 53°C (for ARE II primers), 58°C (for ARE I and *VEGF* V88 primers) and 3) 30 sec extension at 72°C; amplification was completed with a 2 min final extension at 72°C. PCR products were electrophoresed on 1% agarose gel, and ethidium bromide stained DNA was visualized by a gel doc system (Biorad, Hercules, CA).

For quantitation of immunoprecipitated chromatin by quantitative real-time PCR (qRT-PCR), purified DNA samples were amplified in an ABI 7000 thermocycler using primers listed above and following manufacturer’s recommendation for SYBR Green Q-PCR (Applied Biosystems, Foster City, CA). Each PCR reaction was carried out in triplicate and average Ct values were normalized to total input (non- immunoprecipitated) DNA. The amount of DNA immunoprecipitated with the target antibody from hormone treated cells R1881 was compared to that of control samples treated only with vehicle. Shown are input normalized Ct values from chromatin of treated cells relative to untreated control cells.

### RNA isolation and quantitative real-time PCR

RNA was isolated from subconfluent cells using the GenElute Mammalian Total RNA Miniprep Kit (Sigma, St. Louis, MO) as per manufacturer’s recommendation. Following quantitation, 1μg of RNA was reverse transcribed using the High Capacity cDNA ReverseTranscription Kit (Applied Biosystems, Carlsbad, CA). qRT-PCR was performed using either Taqman Universal Master Mix with pre-designed Taqman Gene Expression Assay probe sets for *VEGFA* (Hs00900057_m1) and *18S* (Hs99999901_s1) or SYBR Green Master Mix with primers specific for *VEGF*, *Sp1*, *GAPDH*, and *Beta-actin*: *VEGF* (FOR): 5^′^-CGAAACCATGAACTTTCTGC-3^′^, *VEGF* (REV): 5^′^-CCTCAGTGGGCACACACTCC-3^′^, *Sp1***(**FOR) 5^′^-TGCATTTCAAGGAATGGAAT-3^′^, *Sp1* (REV) 5^′^-GCTTCCTTGGTGTGAAGAGA-3^′^,* GAPDH* (FOR): 5^′^-CCATCACCATCTTCCAGGAG-3^′^, *GAPDH* (REV): 5^′^-GGATGATGTTCTGGAGAGCC-3^′^, *Beta-actin* (FOR): 5^′^-GTGGGGCGCCCCA GGCACCA-3^′^, *Beta-actin* (REV): 5^′^-GTCCTTAATGTCACGCACGATTTC-3^′^). The comparative Ct method [30] was used to analyze gene expression differences between control (untreated) cells and cells treated with R1881 alone or with the anti-androgen casodex.

### Western blot

Subconfluent monolayers of LNCaP and 22Rv1 cells were washed in PBS and proteins were extracted using RIPA lysis buffer (1% NP40, 0.5% Na Deoxycholate, 0.1% SDS, and 150 mM NaCl) containing protease inhibitors. To quantify the amount of proteins present in each lysate, bicinchoninic acid (BCA) assays (Pierce, Thermo Fisher Scientific, Rockford, IL) were performed and absorbance was measured at 600nm on a Dynex Technologies (Chantilly, VA) MRX Revelation plate reader. Proteins (25-50ug) were separated by sodium dodecyl sulfate polyacrylamide gel electrophoresis (SDS-PAGE). After transfer to a Polyvinylidene Fluoride (PVDF) membrane and blocking with 5% casein, blots were probed overnight at 4°C with polyclonal VEGF and AR antibodies (Santa Cruz), and monoclonal β-actin antibody (GenScript). Washed blots were then incubated for 1 hr in either HRP-conjugated anti – rabbit (GenScript) or anti – mouse (Santa Cruz) antibodies. Proteins were visualized by incubating the membrane in a luminol ECL solution followed by chemiluminescent detection using a Fuji LAS 3000 (GE, Piscataway, NJ) detection system. Bands were quantified using ImageJ analysis and normalized to actin levels.

### Co-Immunoprecipitation

Nuclear extracts from 22Rv1 cells were prepared using Active Motif’s Universal Magnetic Co-IP kit (Carlsbad, CA) as per manufacturer’s recommendations. Cells were swelled in Hypotonic Buffer containing phosphatase-, deacetylase-, and protease- inhibitors, then lysed in 5% detergent. This suspension was then centrifuged at 14,000 x g and the supernatant was discarded leaving the nuclear fraction which was enzymatically sheared in the presence of the same inhibitors. Nuclear extracts (150-200μg) were then combined with 5μg of either AR (Santa Cruz) or Sp1 (Upstate) antibodies, or negative control IgG in the presence of the same inhibitors. Following antibody incubation, complexes were pulled down with Protein G magnetic beads. After washing, these complexes were separated by SDS-PAGE and identified by Western blot analysis (as described above).

### Plasmid Transfection and Luciferase Assay

The pGL3-VEGF luciferase reporter constructs (V88, V411, or V2274) were generously provided by Dr. Xie [16] and DNA purified by the Qiagen plasmid Maxi prep kit (Qiagen, Valencia, CA). LNCaP and 22Rv1 cells were plated in 12-well plates as described above. After reaching ~70-90% confluency, cells were serum-starved for 18–24 hours with serum-free RPMI and then transfected with VEGF reporter constructs using Lipofectamine 2000 (Invitrogen, Carlsbad, CA) as described [7]. After 4–6 hours, transfection media was replaced with appropriate growth media. For hormone induction, 5nM R1881 was added to media with 5-10% charcoal-dextran stripped FBS as described above. After 48 hours cells were lysed and luciferase activity was measured using a Promega luciferase assay kit (Promega, Sunnyvale, CA) and a Turner luminometer (Promega, Sunnyvale, CA) following manufacturer’s recommendations. The luciferase activity was normalized to total cell protein, using a micro BCA protein assay, as described above. All experiments were done in triplicate and repeated at least three times. Standard errors of the mean were determined using GraphPad InStat software (San Diego, CA). Significance was determined by Student’s t-test.

### Site directed mutagenesis

Potential binding sites in the *VEGF* promoter were identified using MatInspector, as previously described [11]. Predicted AR and Sp1 binding sites in the *VEGF* promoter construct were then mutated using the QuikChange Site-Directed Mutagenesis Kit (Stratagene, Agilent Technologies, Santa Clara, CA). Primers were designed according to the manufacturer’s suggestions using the QuikChange Primer Design Program. Primers containing the desired mutation (shown in bold) are listed below:

(ARE I (FOR):

5^′^-CTCTATCGATAGGTACCGTG***GTCAG***CTCTCCCC ACCCGTC CCTGTC-3^′^,

ARE I (REV): 5^′^GACAGGGACGGGTGGGGAGAG***CTGAC***CACGGTACCTATCGA TAGAG-3^′^, 

ARE II (FOR): 5^′^-GGAACCACACAGCTTCCC***ACTG***TCAGCTCCACA AAC TTGG-3^′^,

ARE II (REV): 5^′^-CCAAGTTTGTGGAGCTGA***CAGT***GGGAAGCTGTGTGGTTCC-3^′^,

ARE III (FOR): 5^′^-GCCCCAAGATGTCTACAGCTT***ACGG***TCCTGGGGTGC-3^′^,

ARE III (REV): 5^′^-GCA CCCCAGGA***CCGT***AAGCTGTAGACATCTTGGGGC-3^′^,

Sp1.2/Sp1.3 (FOR): 5^′^-GCCCC CCG G***TT*** CGGGCCGGG***TT***CGGGGTCCC-3^′^,

Sp1.2/Sp1.3 (REV): 5^′^-GGGACCCCG ***AA***CC CGG CCC G***AA*** CCGGGGGGC-3^′^,

Sp1.4 (FOR): 5^′^-GGGTCCCGGCGG***TT***CGGAGCCATGCG-3^′^,

Sp1.4 (REV): 5^′^-CGCATGGCTCCG***AA***CCGCCGGGACCC-3^′^).

PCR was performed using the V88, the V411, or the V2274 luciferase reporter constructs and the appropriate mutant primers. After PCR amplification, parental strands were digested with *DpnI* and XL1-Blue super competent cells were transformed with remaining mutant DNA. Individual colonies were grown, plasmids were purified (Qiagen, Valencia, CA) and sequenced to verify that the correct base pairs were changed (Cleveland Clinic Genomics Core, Lerner Research Institute, Cleveland, OH). Luciferase assays were performed using mutant constructs as described above.

## Competing interests

The authors declare that they have no competing interests.

## Authors’ contributions

KE and GF conceived and designed study; analyzed and interpreted data; drafted and revised the manuscript. KE performed experiments and the statistical analyses. CJB and MM assisted with luciferase and qRT-PCR assays. AB assisted with sequence analyses and co-immunoprecipitations. All authors read and approved the manuscript.

## Supplementary Material

Additional file 1 Figure S1 R1881 (1nM) induces VEGF mRNA expression in LNCaP cells. LNCaP cells were serum starved overnight followed by treatment with either 0nM R1881 (DMSO) or 1nM R1881 for 48 hours. VEGF mRNA expression was measured by qRT-PCR and normalized to 18S levels as described. Values represent fold change relative to DMSO treatment. A Student’s t-test was performed and significance was determined ** (p < 0.01). Click here for file

Additional file 2 Figure S2 Mutation of the Sp1.4 binding site does not eliminate basal activity of the VEGF core promoter. 22Rv1 cells were transfected with mSp1.4 (in V88 core promoter construct) or pGL3- Basic empty vector. Cells were transfected and luciferase assays were performed as described. Experiments were performed in triplicate and repeated twice. Luciferase activity is shown relative to average normalized activity of the pGL3-Basic empty vector. Significance was determined by Student’s t-test *** (p < 0.001). Click here for file
