# Supplementary figures and images for: Androgen up-regulates vascular endothelial growth factor expression in prostate cancer cells via an Sp1 binding site
Source: Mol Cancer. 2013 Feb 1;12:7. doi: 10.1186/1476-4598-12-7 (PMC3616929; doi:10.1186/1476-4598-12-7)

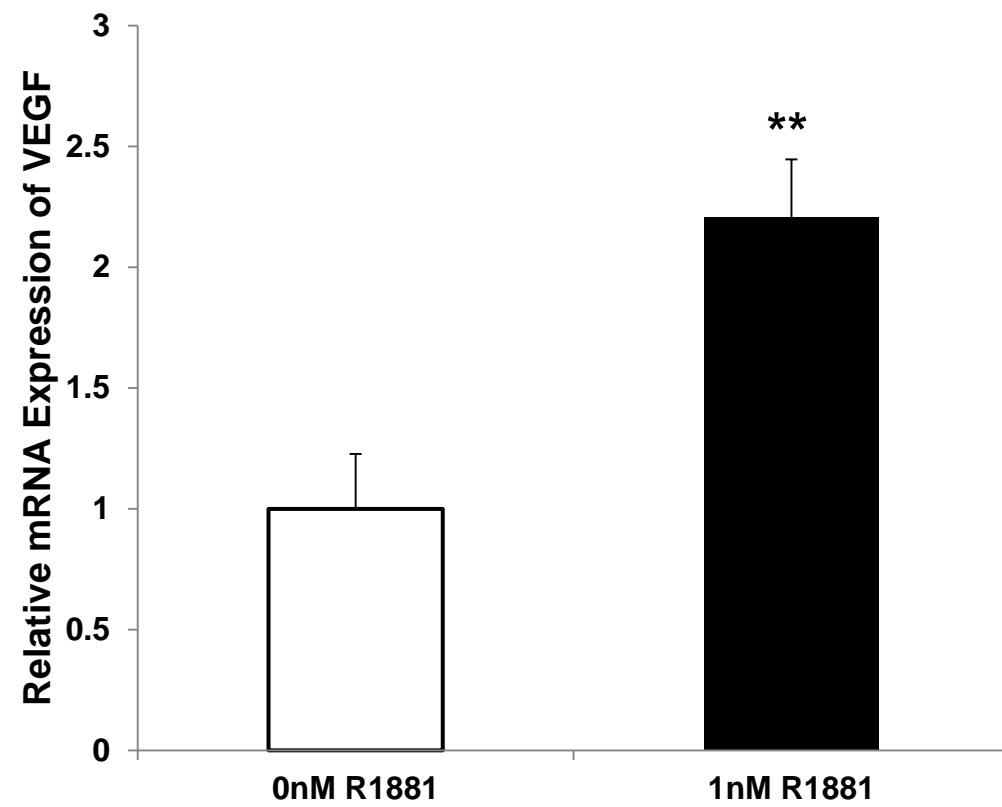

Supplement: Additional file 1 Figure S1 — R1881 (1nM) induces VEGF mRNA expression in LNCaP cells. LNCaP cells were serum starved overnight followed by treatment with either 0nM R1881 (DMSO) or 1nM R1881 for 48 hours. VEGF mRNA expression was measured by qRT-PCR and normalized to 18S levels as described. Values represent fold change relative to DMSO treatment. A Student’s t-test was performed and significance was determined ** (p < 0.01). [file 1476-4598-12-7-S1.pdf]

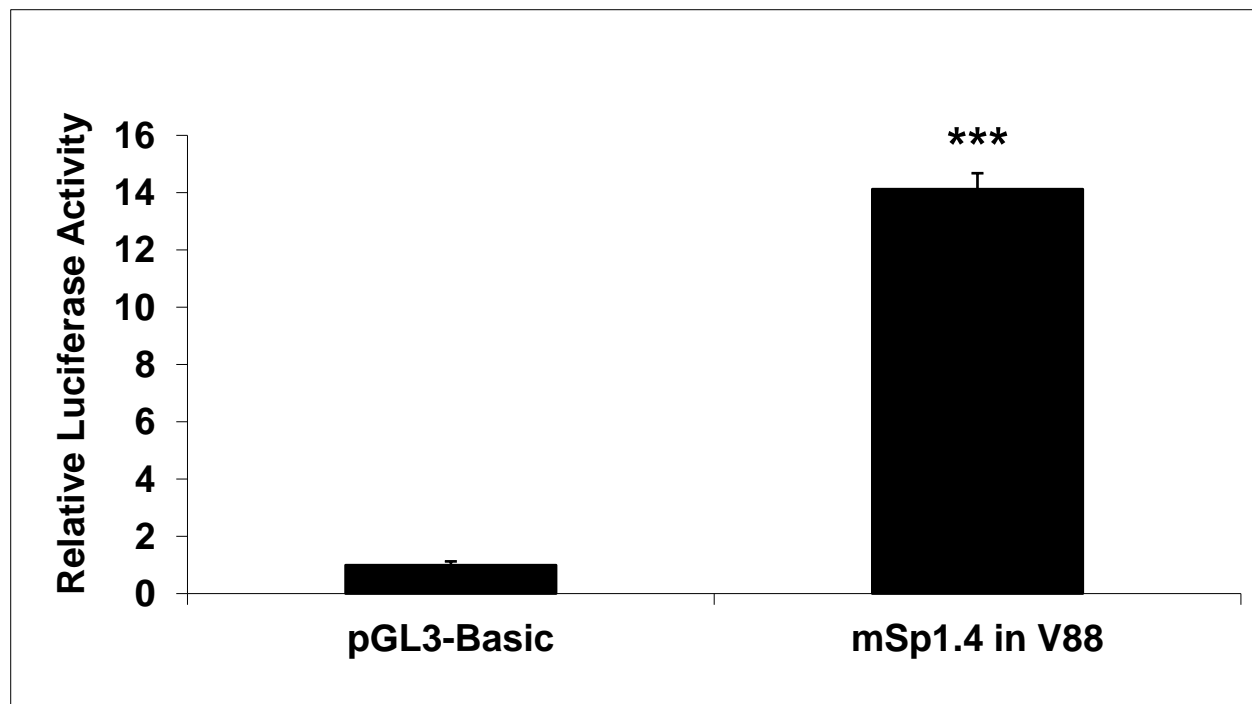

Supplement: Additional file 2 Figure S2 — Mutation of the Sp1.4 binding site does not eliminate basal activity of the VEGF core promoter. 22Rv1 cells were transfected with mSp1.4 (in V88 core promoter construct) or pGL3- Basic empty vector. Cells were transfected and luciferase assays were performed as described. Experiments were performed in triplicate and repeated twice. Luciferase activity is shown relative to average normalized activity of the pGL3-Basic empty vector. Significance was determined by Student’s t-test *** (p < 0.001). [file 1476-4598-12-7-S2.pdf]
